# Combined rotation scarf and Akin osteotomies for hallux valgus: a patient focussed 9 year follow up of 50 patients

**DOI:** 10.1186/1757-1146-3-2

**Published:** 2010-02-15

**Authors:** Timothy E Kilmartin, Claire O'Kane

**Affiliations:** 1 Hillsborough Private Clinic, Hillsborough, Co Down, Northern Ireland BT26 6AE, UK; 2 North West Independent Hospital, Ballykelly, Co Derry, Northern Ireland BT49 9HS, UK; 3 Department of Podiatric Surgery, Derbyshire County Primary Care Trust, Ilkeston Hospital, Heanor Road, Ilkeston Derbyshire DE7 8LN, UK

## Abstract

**Background:**

The Cochrane review of hallux valgus surgery has disputed the scientific validity of hallux valgus research. Scoring systems and surrogate measures such as x-ray angles are commonly reported at just one year post operatively but these are of dubious relevance to the patient. In this study we extended the follow up to a minimum of 8 years and sought to address patient specific concerns with hallux valgus surgery. The long term follow up also allowed a comprehensive review of the complications associated with the combined rotation scarf and Akin osteotomies.

**Methods:**

Between 1996 and 1999, 101 patients underwent rotation scarf and Akin osteotomies for the treatment of hallux valgus. All patients were contacted and asked to participate in this study. 50 female participants were available allowing review of 73 procedures. The average follow up was over 9 years and the average age at the time of surgery was 57. The participants were physically examined and interviewed.

**Results:**

Post-operatively, in 86% of the participants there were no footwear restrictions. Stiffness of the first metatarsophalangeal joint was reported in 8% (6 feet); 10% were unhappy with the cosmetic appearance of their feet, 3 feet had hallux varus, and 2 feet had recurrent hallux valgus. There were no foot-related activity restrictions in 92% of the group. Metatarsalgia occurred in 4% (3 feet). 96% were better than before surgery and 88% were completely satisfied with their post-operative result. Hallux varus was the greatest single cause of dissatisfaction. The most common adverse event in the study was internal fixation irritation. Hallux valgus surgery is not without risk and these findings could be useful in the informed consent process.

**Conclusions:**

When combined the rotation scarf and Akin osteotomies are an effective treatment for hallux valgus that achieves good long-term correction with a low incidence of recurrence, footwear restriction or metatarsalgia. The nature of the osteotomies allows early return to normal shoes and activity without the need for postoperative immobilisation in a plaster cast.

## Introduction

The Cochrane review of hallux valgus surgery has disputed the scientific validity of hallux valgus research [1]. The review reported that although many studies were available on the surgical management of the condition, final outcome measures were most frequently measured at one year with just a few trials maintaining follow up for 3 years. Scoring systems and surrogate measures such as x-ray measurements were commonly used but these were considered of dubious relevance to the patient if they did not address their main concerns. In all the literature considered by the Cochrane review, just one study asked the patients if they were better than before surgery [2]. The review recommended that future research should include patient focussed outcomes and follow up periods of at least 5 to 10 years.

In reviewing hallux valgus surgical outcomes it is notable that a high proportion of patients, 25-33%, remain dissatisfied at final follow up [1]. Schneider and Knahr reviewed the expectations of both patients and surgeons in hallux valgus surgery [3]. Two hundred patients were interviewed and their principal concern was relief of foot pain when wearing a conventional shoe. Importantly, the patients hoped that surgery would restore unlimited pain free walking, whereas alignment and cosmesis of the hallux was considered of little importance by either surgeons or patients. When the surgeons were interviewed (186 surgeons of the German Austrian Orthopaedic Foot Surgery Society), their primary concern was also pain and shoe fitting issues but in addition restoring adequate range of motion to the first MTP joint and relieving metatarsalgia.

Common complications specific to hallux valgus surgery include recurrence of deformity, first metatarsophalangeal (MTP) joint stiffness and transfer metatarsalgia [4]. With the exception of recurrence, it is unlikely that any of these known postoperative complications will be of automatic concern to the patient prior to surgery. Their occurrence could, however, explain the high levels of postoperative dissatisfaction even when hallux valgus angles and first MTP joint pain have improved with surgery [1]. While many previous studies have focussed on x-ray outcomes, the prevalence of these specific complications provides a more patient focussed measure of the outcome of a particular procedure and will help surgeons prepare the patients for informed consent.

The scarf osteotomy was first developed in 1926 by Meyer but never achieved widespread use due to inadequate fixation techniques [5]. Weil popularised the technique after describing an effective fixation technique using two AO screws [6,7]. The advantages of the technique included: rigid compression of large areas of bone to bone contact providing a good environment for primary bone healing and early return to normal weight bearing activities and range of motion exercises preventing joint stiffness and oedema [8]. The scarf osteotomy also avoided the complication of metatarsus elevatus associated with more proximal metatarsal osteotomies [9], allowed accurate correction of the intermetatarsal angle and could be modified to allow the metatarsal to be shortened or lengthened, and plantarly or dorsally displaced if required [10].

**Figure 1:**
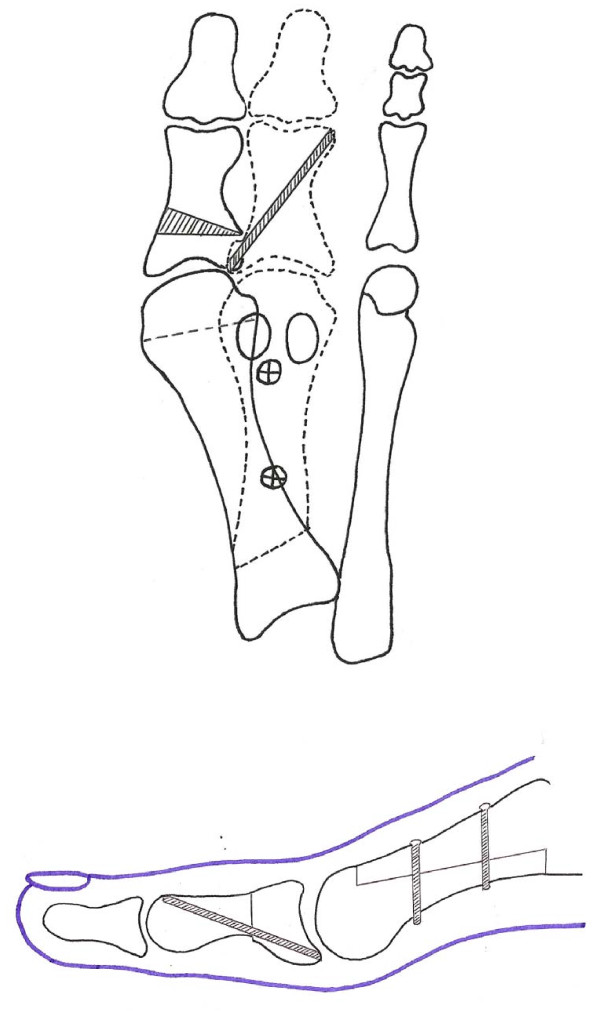
**The rotation scarf osteotomy rotates the inferior fragment as opposed to transposing it in the scarf ostoeotomy**. By rotating the fragments greater reduction of the intermetatarsal angle can be achieved and the cortices of the metatarsal fragments are crossed preventing troughing.

The scarf osteotomy has been extensively reviewed in recent literature [10-19]. To date the scarf has generally been used to correct moderate hallux valgus in the presence of intermetatarsal angles of less than 15 degrees, the limiting factor being that if the inferior fragment is transposed too far laterally, fixation cannot be obtained and there will be insufficient bone to bone contact to produce stable union of the osteotomy. Thus the scarf osteotomy may not be indicated in the treatment of severe hallux valgus with high intermetatarsal angles. This is frustrating for the foot surgeon as all the advantages of the scarf osteotomy cannot be applied to patients with more severe deformity. In view of the limitations of the scarf osteotomy, Duke modified the procedure and introduced the rotation scarf osteotomy [20] (Figure 1). This osteotomy is able to reduce higher intermetatarsal angles, while maintaining excellent stability, thereby avoiding the complications and extended recovery time associated with more proximal osteotomies or arthrodesis. Another significant advantage of the rotation scarf osteotomy is that crossing the cortices prevents 'troughing', a known complication of the transpositional scarf osteotomy which can lead to elevatus of the metatarsal head [14].

In this study we attempted to learn more about the patient's satisfaction with their surgical outcome as well as the incidence of common complications and their long term impact on the patient. We reviewed 50 cases (73 feet) and specifically asked participants if they were better after their hallux valgus surgery. We also assessed them for transfer metatarsalgia and first MTP joint stiffness. Finally, we asked participants to report any footwear fitting difficulties. In this way we hoped to provide further information for patients on the risks and complications specific to the rotation scarf and Akin osteotomies to enable a more comprehensive informed consent.

## Methods

Between 1996 and 1999, 101 patients underwent combined rotation scarf and Akin osteotomies for the treatment of hallux valgus. In all cases the procedure was performed by the primary author. All patients were contacted and asked to participate in this study which was approved by the local Audit committee. 53 patients returned to be involved in this study. 10 other patients were deceased, 24 were lost to follow up and 14 refused to attend for review but were contacted by telephone and participated in a brief telephone interview.

Of the 53 patients who returned for the study, 3 were excluded (1 was suffering from multiple sclerosis, 1 from rheumatoid arthritis and 1 had undergone revision surgery). Of the 50 participants included, all were female. 23 participants had undergone bilateral surgery so a total of 73 feet were analysed. The average age at the time of surgery was 57, (SD 10) and the average follow up was 9 years 5 months (113 months, SD 11). The clinical review was performed independently by the second author who had not previously been involved in the initial surgical care of the participants.

**Figure 2:**
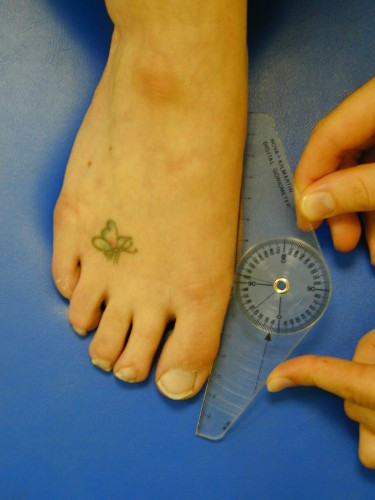
**Goniometric measurement of the hallux valgus angle using a digital goniometer (available from Nova Instruments, Mill House, Newgatestreet Road, Goffs Oak, Herts. EN7 5RX)**.

**Figure 3:**
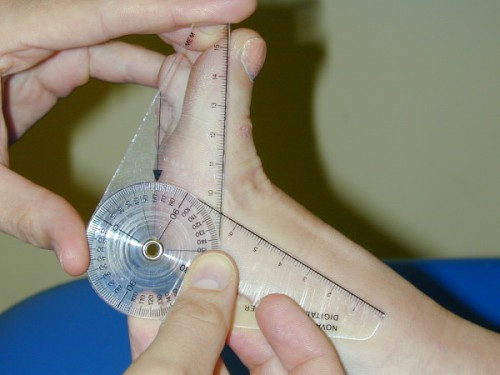
**With the resting non-weightbearing position being considered the zero degree angle, the passive hallux dorsiflexion range of motion was measured using the digital goniometer**.

**Figure 4:**
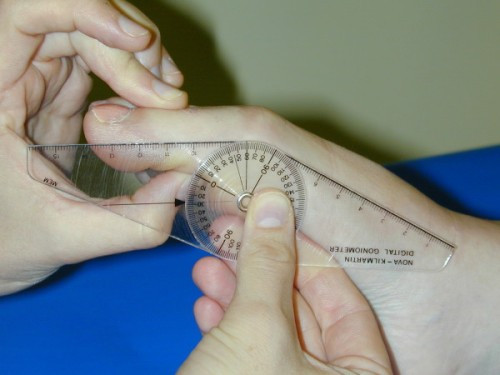
**Passive hallux plantarflexion range of motion was measured from the resting position which was considered zero degrees**.

Preoperatively the first-second intermetatarsal angle and hallux valgus angle were measured on weightbearing bilateral x-rays. The x-ray tube was directed 15 degrees from the vertical in the dorso-plantar direction. The beam was centred on the navicular with a focal distance of 100 cm. Postoperatively the first MTP joint/hallux valgus angle was measured using a digital goniometer (Figure 2), as ethical approval for further irradiation of the participants was not forthcoming. Intra-observer repeatability of the goniometer had previously been established [21]. A good correlation (r = 0.63) between x-ray measurement and goniometric measurement has previously been found [22]. The range of dorsiflexion and plantarflexion of the MTP joint was also assessed using the digital goniometer (Figures 3 and 4).

All the participants were then interviewed and asked if they were completely satisfied, satisfied with reservations or dissatisfied with the results of their surgery. Restrictions with footwear, or any activity restrictions because of their feet were recorded. The participants were asked if there was any pain or stiffness in the first MTP joint. Any pain or tenderness of the lesser MTP joints was also recorded. Finally, the participants were asked if they were happy with the appearance of their post surgical foot and would they be happy to undergo surgery under similar circumstances in the future.

A number of adjunctive procedures were performed. In 22 feet a second toe proximal interphalangeal joint (PIPJ) arthroplasty was performed and in 9 feet PIPJ arthroplasties of other toes were performed. 4 feet underwent a Weil osteotomy of the second metatarsal and 3 feet had neuroma excision from the third intermetatarsal space. With the exception of 1 participant who underwent an adjunctive second joint fusion, all participants were encouraged to return to lace-up or running shoes at 2 weeks postoperatively. Between 4 and 6 weeks off work and sport was recommended.

### Surgical technique

The procedure was performed in all cases under local anaesthetic ankle block on a day case basis. An ankle tourniquet was applied and a medial plantar skin incision running from the interphalangeal joint of the hallux to the base of the first metatarsal was made. This was deepened to the capsule ensuring adequate haemostasis. The capsular incision was made as a double semi-elliptical incision and the ellipse of tissue excised.

**Figure 5:**
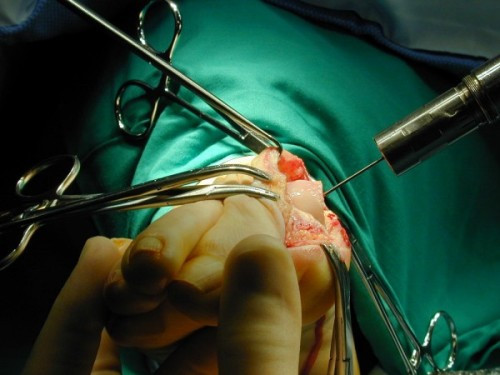
**Placement of the guide wire to achieve plantar displacement of the metatarsal head with the osteotomy**.

**Figure 6:**
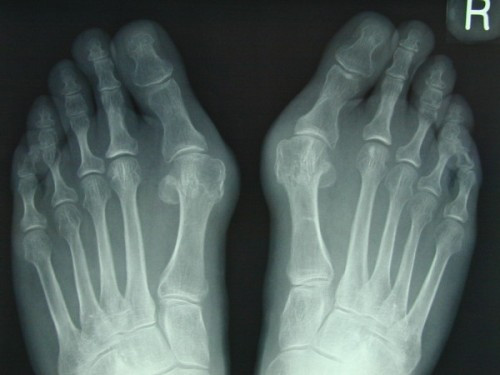
**Rotation scarf and Akin osteotomies pre-operative x-ray**.

**Figure 7:**
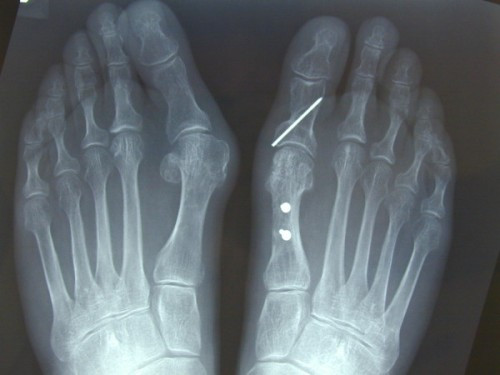
**Postoperative x-ray of the rotation scarf and Akin osteotomies in the right foot**. This x-ray demonstrates fixation of the osteotomy, realignment of the sesamoids and reduction of the intermetatarsal and hallux valgus angle while preserving the length of the metatarsal.

A beaver blade was introduced into the joint capsule between the metatarsal head and the sesamoid apparatus and the adductor hallucis tendon and lateral sesamoid ligament were released from their respective insertions in the metatarsal head and proximal phalanx. The medial eminence of the first metatarsal was resected at the sagittal groove. A guide wire was placed just proximal to the metatarsal head articular surface and just inferior to the first metatarsal dorsal cortex. The guide wire was directed plantarly in the direction of the plantar surface of the third metatarsal head but perpendicular to the long axis of the second metatarsal (Figure 5). An osteotomy guide was placed on the guide wire and a power saw was then used to make the horizontal cut along the metatarsal shaft extending from just proximal to the articular surface of the metatarsal head to the basal tuberosity. The distal cut was made parallel with the guide wire and the proximal cut at approximately a 45° angle from medial proximal to lateral distal in order to allow the rotation to occur (Figure 1). While it is possible to shorten the metatarsal by angling the distal cut in a proximal lateral direction, this was avoided as we consider any loss of first metatarsal length a predisposition to transfer metatarsalgia. The lateral capsule was then released and the inferior fragment rotated toward the second metatarsal to reduce the intermetatarsal angle. The degree of rotation required was established pre operatively by measuring the intermetatarsal angle on x-ray. We aimed to reduce the intermetatarsal angle to 7°. One mm of rotation equals 1° of correction which could be measured by the amount of overhanging bone of the superior fragment once the metatarsal head was rotated. The bone fragments were held with a scarf clamp and fixed with two 2.0 cortical screws using AO technique (Figures 6 and 7). The overhanging edges of bone were then removed from the medial side of the metatarsal shaft. An Akin closing wedge osteotomy of the proximal phalanx was performed on all cases. The Akin osteotomy was fixated using a single 1.2 mm threaded k-wire (Figure 7).

The capsule was then closed using 2-0 vicryl, figure of 8 sutures. The hallux was held in a plantarflexed position as the capsule was closed [10]. As an ellipse of capsule had previously been excised, closing the capsule pulled the sesamoids into a corrected position under the first metatarsal head. Tension on the capsular sutures was increased to further draw the hallux into correction if necessary, though we believe that soft tissue correction is largely temporary and correction should be achieved almost exclusively with the osteotomies. Skin was then closed using 5-0 vicryl subcuticular sutures.

**Figure 8:**
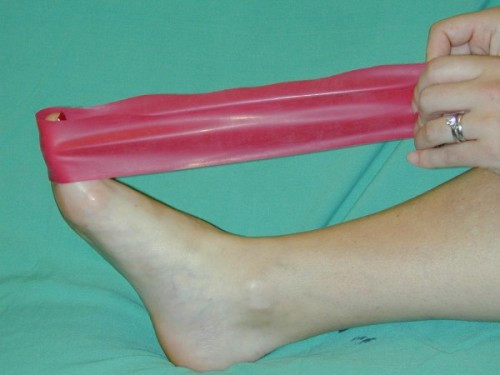
**Post operative flexion exercises using a powerband**. The patient is asked to repeatedly plantarflex the hallux while increasing the resistance of the powerband.

Postoperatively all but one patient who underwent a simultaneous second metatarso-cuneiform joint fusion wore a surgical shoe and used crutches for two weeks. After two weeks, dressings were removed and the participants were encouraged to wear lace up or running shoes and begin returning to normal activities. The participants were advised to perform range of motion exercises against the resistance of a powerband. In particular flexion exercises were encouraged to restore flexor power to the hallux. (Figure 8). We also advised the participants to walk through the hallux on gait. These measures, we believe, may contribute to reducing the risk of transfer metatarsalgia.

## Results

### Patient reported outcomes

In the 50 participants (73 feet) available for follow up 88% of the group (44 participants), were completely satisfied, 8% (4 participants) were satisfied with reservations and 4% (2 participants) were dissatisfied (Table 1). 96% (48 participants) were better than before surgery and 4% (2 participants) were no better. All but one of the study group indicated that they would be happy to undergo surgery again under similar circumstances. 90% of cases (66 feet) were happy with the cosmetic appearance. 10% (7 feet) were unhappy with the cosmetic appearance, 3 had hallux varus and 2 had recurrent hallux valgus. 2 participants felt their feet were still too wide.

**Table 1:** Summary of outcomes for the 50 female participants (73 feet) at an average 9.5 years postoperative rotation scarf and Akin osteotomies for hallux valgus

Outcome	Percentage
Patient satisfaction	88% completely satisfied 8% satisfied with reservations 4% dissatisfied

Cosmetic appearance	10% unhappy with cosmetic appearance

Footwear restrictions	14% could not wear high heels

Goniometric measurement of first MTP joint post op	Mean Hallux valgus angle 10° SD 6 Mean dorsiflexion 54° SD 4.6 Mean plantarflexion 15° SD 8

First MTP joint stiffness	8%

Physical activity restriction	8%

Metatarsalgia	6%

First MTP joint pain	6%

Hallux valgus recurrence	8%

Hallux varus	4%

Post op superficial wound infection	4%

Internal fixation removal	25%

Revision surgery	1 patient for hallux varus

There were no activity restrictions in 92% of the group (46 participants). Walking distance was restricted to less than 3 miles in 2 participants. 1 participant felt she could no longer do yoga because of first MTP joint stiffness and 1 participant had developed midfoot arthritis which was causing activity restriction due to pain. In 94% of the group (69 feet), there was no metatarsalgia. Metatarsalgia occurred post operatively in 4% of the group (3 feet), all of these had hallux varus. 1 participant had metatarsalgia prior to surgery and this was still present postoperatively.

### Footwear issues

In 86% of the sample (63 feet) there were no footwear restrictions. High heels could not be accommodated in 14% (10 feet). This restriction was attributable to surgery in 7% of the sample (5 feet) where there was postoperative first MTP joint stiffness. In one other case internal fixation irritation was restricting the use of court style shoes. Hallux varus, which had developed postoperatively, was causing footwear problems to 1 participant, and metatarsalgia, which had developed postoperatively, was restricting the use of thin-soled fashion shoes in 1 case. Two participants had developed hammer toe deformities of the 2^nd ^digit that restricted shoes.

### Joint alignment, range of motion and pain

Preoperatively the mean hallux valgus angle measured on weight bearing bilateral x-rays was 37 degrees (SD 7). The mean first-second intermetatarsal angle was 16 degrees (SD 3). At final follow-up the goniometric measurement of the first MTP joint/hallux valgus angle was 10 degrees (SD 6). The mean dorsiflexion at the first MTP joint was 54 degrees (SD 14.6) and the mean plantarflexion 15 degrees (SD 8) - normal ranges are reported to be 65 to 90 degrees dorsiflexion and 15 to 20 degrees plantarflexion [23-25].

Hallux valgus recurrence with first MTP joint/hallux valgus angles in excess of 15 degrees was noted in 8% of the sample (6 feet). In 2 participants the hallux valgus angle was 22 degrees and in 4 participants it was 20 degrees. Hallux varus occurred in 4% (3 feet). Postoperative soft tissue infection managed with oral antibiotics occurred in 4% of the sample (3 feet). 1 participant required revision surgery for hallux varus and 25% of the sample (18 feet) required removal of the distal metatarsal screw.

No stiffness of the first MTP joint was reported by 92 percent of the sample (67 feet). First MTP joint stiffness occurred in 8% (6 feet) and in 5 feet this caused footwear restrictions. In this subset, the mean dorsiflexion was 46 degrees (SD 19, range 22 to 74 degrees) and the mean plantarflexion was 10 degrees (SD 1.6, range 0 to 10 degrees). In 94% of the group (69 feet), there was no first MTP joint pain. First MTP joint pain was present in 3% of the group (two feet) and in both cases there was hallux varus. In 2 other feet there was occasional joint pain.

None of the 14 participants contacted by telephone had required revision surgery at other facilities. All were happy with the outcome of their surgery. No further information was gathered from these telephone interviews.

## Discussion

In the original cohort of 101 patients undergoing the combined rotation scarf and Akin osteotomies 98% were female. All 50 participants that returned for assessment related to this study were female. The higher incidence of symptomatic hallux valgus in females is well documented [26,27], but there is far less consideration of what drives female patients to undergo surgery and what their expectations of surgery are [3]. Hallux valgus is often caused by shoe fitting issues wherein many of the symptoms are caused by footwear irritation and the expectations of surgery are a return to a wide range of shoe styles which previously have been difficult [3]. In this context, hallux valgus surgery could be seen as a high risk intervention because although it may allow easier footwear accommodation, it carries the possibility of rendering the foot painful due to the specific complications of first MTP joint pain and stiffness and transfer metatarsalgia. Recurrence of hallux valgus is also a disappointing outcome for many patients [28], because once again it recreates the shoe fitting problems.

Foot surgeons may find it difficult to accept the possibility that they could be performing hallux valgus correction for cosmetic reasons but female interest in fashionable, high-heeled footwear is high. In this series of participants we believe we only performed surgery when conservative measures failed to alleviate symptoms or when participants could not accommodate their foot in conventional shoes, or when the hallux was so malaligned that it was beginning to underide the second toe and deform previously normal structures within the foot. On the basis of Schneider and Khnar's study [3], we recognise the importance of footwear postoperatively and fixed on this as a patient focussed outcome.

At an average of 9.5 years after their operation, 86% of the sample were unrestricted in their footwear choice in that they could wear high heels. Patients that can wear high heeled shoes comfortably are unlikely to be suffering from painful first MTP joint stiffness or from transfer metatarsalgia. In this way the ability to wear a range of shoes is also an indication of foot function. In this sample just 4% were found to be suffering from transfer metatarsalgia, but 8% were aware of first MTP joint stiffness and in 6% there was joint pain.

The management of transfer metatarsalgia and first MTP joint stiffness following hallux valgus correction has received little attention in the literature and is certainly an area with much potential for further investigation. We sought to prevent both problems by asking patients to mobilise and strengthen the first MTP joint immediately postoperatively with simple flexions of the first MTP joint. At two weeks postoperatively we asked patients to use a powerband (rubber band exerciser) to perform plantarflexion and dorsiflexion exercise of the first MTP joint against the resistance of the powerband. We also advised patients to propel through the first MTP joint and hallux on gait so as to avoid guarding the first MTP joint. If the hallux cannot be plantarflexed, propulsion power from the hallux is reduced and we believe the patient is more likely to propel from the lesser MTP joints, which then become bruised, inflamed and painful. Intraoperatively we always attempted to maintain the length of the first metatarsal and displace the metatarsal head in a plantarly direction as part of the rotation scarf osteotomy. This again, we believe, may minimise the possibility of transfer metatarsalgia.

Recurrence of hallux valgus occurred in 8% of the participants in this study. This is a disappointing outcome as it means the patient is once more at risk of developing the whole range of symptoms associated with hallux valgus. However, cases of recurrent hallux valgus were considered mild as a maximum hallux valgus angle of 22 degrees was observed. This is close to the normal reported range of 15 degrees or less [29].

Hallux varus developed in just 3 feet but at interview these participants appeared more unhappy with their outcome than any other participant in the study. We consider hallux varus a significant though rare complication leading to progressive joint degeneration and pain, metatarsalgia and footwear fitting problems. Its real significance lies in the degree of dissatisfaction it creates with patients often presenting with multiple symptoms. Hallux varus occurs when the tibial sesamoid is positioned medial to the first metatarsal head [30-32]. In the rotation scarf and Akin osteotomies hallux varus may be a consequence of excessive reduction of the intermetatarsal angle by the metatarsal osteotomy. Alternatively, excessive mobilisation of the sesamoids following detachment of the fibular sesamoid suspensory ligament, especially when combined with release of the adductor hallucis tendon, will risk hallux varus. Over tightening the medial capsule during deep closure will compound this effect by pulling the tibial sesamoid medial to the metatarsal groove. An excessively aggressive Akin osteotomy will also pull the hallux into varus. Of all these potential causes of hallux varus, the Akin osteotomy is the easiest to assess intraoperatively and certainly if the hallux appeared in varus after performing the Akin osteotomy, the wedge of bone would be re-inserted and the osteotomy fixed. The position of the tibial sesamoid was also assessed intraoperatively and if it was not sitting directly inferior to the medial sesamoid groove, the rotation of the inferior fragment would be reduced before internal fixation was performed. Over tightening of the medial capsule will pull the hallux into varus as the capsule is sutured. Sutures can be removed at this point, a smaller bite of the capsule taken and less tension applied to the suture.

Clearly, in the three cases of hallux varus in this study one or all of these predisposing factors continued to malalign the MTP joint. This complication, however, must be considered alongside the relatively low incidence of hallux valgus recurrence, which we believe is a consequence of the ability of the rotation scarf and Akin osteotomies to address all components of the hallux valgus deformity. In particular, we believe addressing the position of the hallux with the Akin osteotomy is vital to ensure that the hallux lies parallel but not abutting the second toe. Pressure of the hallux against the second toe will cause the proximal phalanx to act like a wedge driving the first metatarsal once more into varus [33].

The place for the Akin osteotomy in combination with first metatarsal osteotomy is increasingly acknowledged in the literature [10,34,35]. Traditionally, however, hallux valgus repair involved osteotomy of the first metatarsal only. The position of the hallux improved as a consequence of reducing the metatarsus primus varus, realigning the sesamoids, and crucially, shortening the first metatarsal, which relaxed the soft tissue contractions around the MTP joint and in effect, allowed the hallux to 'spring' straight [3]. In contrast, the rotation scarf osteotomy used in this study did not shorten the first metatarsal and hence the hallux position was addressed separately by the Akin osteotomy. The Akin osteotomy allows a very deliberate and controllable correction of the hallux position and its use in combination with the rotation scarf probably explains why recurrence of hallux valgus, an important cause of patient dissatisfaction in most hallux valgus surgery studies and a universal finding in one long-term follow up study of the Mitchell osteotomy [28], occurred in just 6 feet in this study of 73 hallux valgus corrections.

The most common adverse event in the study was internal fixation irritation. One quarter of the participants required removal of the distal screw from the metatarsal shaft due to footwear irritation. In most cases the participants found that the distal screw was irritated by the proximal edge of the toe box in court style shoes. Currently, the distal screw is now countersunk more aggressively and placed as proximal on the metatarsal shaft as possible to achieve the greatest depth of soft tissue coverage and reduce proximity to the shoe toe box.

In this study we evaluated the long-term outcomes of the rotation scarf and Akin osteotomies to treat participants with severe hallux valgus associated with high intermetatarsal angles usually in excess of 15 degrees [36]. Normally in these circumstances more proximal osteotomies or indeed fusions of the first metatarsocuneiform joint are recommended [36]. The advantages of the rotation scarf and Akin osteotomies over the scarf osteotomy alone or more proximal metatarsal osteotomies or fusions include:

(i) Accurate correction of the intermetatarsal angle, 1 mm of lateral transposition of the metatarsal head equals 1° of intermetatarsal angle correction allowing intraoperative evaluation of the correction [37].

(ii) Large areas of bone to bone contact reducing the risk of non union.

(iii) High stability of the osteotomy with the inferior and superior fragments of the metatarsal being pushed together by weight bearing forces rather than being distracted apart by those forces, which may happen in the Lapidus first metatarsocuneiform fusion or oblique closing wedge osteotomy procedures. The 2 points of fixation also contributes to quick return to normal weightbearing activities at 2 weeks postoperatively, thus decreasing the risk of complications associated with prolonged immobilisation of the postoperative patient.

(iv) Rotation of the inferior fragment also allows greater correction of the intermetatarsal angle than can be achieved with the scarf osteotomy, where the inferior fragment can be transposed no more than 50% of the width of the superior fragment.

(v) Troughing of the fragments is an important risk with the transposition scarf [14]. Because the rotation scarf osteotomy causes the cortical shell of the dorsal fragment to form an 'X' shape with the plantar fragment (Figure 1), troughing is prevented.

The results of this study need to be viewed in light of a number of limitations. While this study sought to establish participants' views of their hallux valgus surgery outcome it could have been strengthened by the use of validated patient-reported outcome measures, such as the Manchester Oxford Foot Questionnaire [27]. Unfortunately, this become available after most participants received their initial surgery and it cannot be validly applied retrospectively.

Clearly, the postoperative goniometric measurement of first MTP joint angles rather than the x-ray measurement does weaken this study. However, ethical requirements in the future may prevent x-rays being used as an outcome measure in postoperative outcome studies. The validity of first MTP joint/hallux valgus angle measurements using the digital goniometer has been evaluated in a study of 77 childrens' feet with hallux valgus, who underwent weightbearing x-ray and charting of the first MTP joint angle followed by digital goniometric measurement [22]. The correlation between the two methods was good (r = 0.63). The mean angle charted on x-ray was 20 degrees (SD 4) and the mean angle measured with the goniometer was 19 degrees (SD 4). This was, however, a statistically significant difference (p < 0.05), with the goniometer generating on average a 1° smaller value (SD 3.5), the 95% confidence interval being 0.4 to 2 degrees. This very small difference in measurement does support the use of the digital goniometer and we would recommend it for similar outcome studies where use of x-rays are prohibited by an ethical committee.

Severe hallux valgus is a syndrome that involves the whole forefoot [36]. While it is not surprising that many adjunctive forefoot procedures were performed in this group with marked hallux valgus deformity, the additional procedures could potentially have confounded our attempts to measure the outcome of the hallux valgus surgery only. In all cases, however, the participants were asked to focus exclusively on the surgery to their great toe in the responses they provided.

The rotation scarf and Akin osteotomies are an effective treatment for hallux valgus. It achieves good long term correction with a low incidence of recurrence, joint stiffness, or metatarsalgia. Hallux varus was the single most important cause of patient dissatisfaction. The nature of the osteotomy allows the surgeon flexibility to correct a range of positional abnormalities within the first ray while allowing early return to normal shoes and activity without the need for postoperative immobilisation in a plaster cast.

## Competing interests

The authors declare that they have no competing interests.

## Authors' contributions

TEK performed all the surgery, assisted in design of the study, analysed and summarized the data and drafted the manuscript. CO designed the study, reviewed all the participants, collected the data and assisted in data analysis. Both authors read and approved the final manuscript.
